# Supplementary material for: Content-rich biological network constructed by mining PubMed abstracts
Source: BMC Bioinformatics. 2004 Oct 8;5:147. doi: 10.1186/1471-2105-5-147 (PMC528731; doi:10.1186/1471-2105-5-147)
Supplement: Additional File 2 — The original results of the above study (non-essential files are deleted to keep the file size under the limit set by BMC bioinformatics). [file 1471-2105-5-147-S2.bz2 › chilibotAdditionalFile2/dip40/325ID8679714E3498/html/NCF4_NCF1.html]

 


 **NCF4** and **NCF1** 
  
Found 79 abstracts in PubMed, retrieved 40.  
 

 What does Google say? 
 PDF only 
| .edu only 

---

**Interactive relationship** (e.g. stimulation, inhibition, etc)

**Stimulatory relationship**- Here we show that p40 phox  [ **NCF4** ]  enhances membrane translocation of p67 phox and p47 phox  [ **NCF1** ]  in stimulated cells, which leads to facilitated production of superoxide.  Ref: 12456638 EMBO J, 2002
- p40 phox  [ **NCF4** ]  Participates in the activation of NADPH oxidase by increasing the affinity of p47 phox  [ **NCF1** ]  for flavocytochrome b 558.  Ref: 10861218 Biochem J, 2000
- Data are presented here using the cell free system for NADPH oxidase activation that shows that p40 phox  [ **NCF4** ]  can function to promote oxidase activation by increasing the affinity of p47 phox  [ **NCF1** ]  for the enzyme approx.  Ref: 10861218 Biochem J, 2000
**Neutral relationship**- The PX domains of p47phox  [ **NCF1** ]  and p40phox  [ **NCF4** ]  bind to lipid products of PI 3 K.  Ref: 11433300 Nat Cell Biol, 2001
- We show here that the PX domains in p47phox  [ **NCF1** ]  and p40phox  [ **NCF4** ]  subunits of the phagocyte NADPH oxidase bind to phosphatidylinositol bisphosphate PtdIns P 2 and phosphatidylinositol 3 phosphate PtdIns 3 P, respectively.  Ref: 11433300 Nat Cell Biol, 2001
- Furthermore, we show that its N terminal part binds to the PX domain of p47phox  [ **NCF1** ]  and p40phox  [ **NCF4** ] .  Ref: 11716484 Biochem Biophys Res Commun, 2001
- The NADPH oxidase components p47 phox  [ **NCF1** ]  and p40 phox  [ **NCF4** ]  bind to moesin through their PX domain.  Ref: 11716484 Biochem Biophys Res Commun, 2001
- Moesin belongs to the ezrin radixin moesin family of F actin binding proteins and we show that it binds to p47phox  [ **NCF1** ]  and p40phox  [ **NCF4** ]  in a phosphoinositide dependent manner.  Ref: 11716484 Biochem Biophys Res Commun, 2001
- The Vam7p PX domain has the same overall alpha beta fold observed in the structures of the ligand free p47 phox  [ **NCF1** ]  PX domain and the PtdIns 3 P bound p40 phox  [ **NCF4** ]  PX domain, exhibiting several similarities and differences with these two PX domains.  Ref: 11993989 Biochemistry, 2002
- Analysis of the p47 phox  [ **NCF1** ]  and p67 phox amino acid sequences showed two potential Src homology three domains and p40 phox  [ **NCF4** ]  one.  Ref: 11182145 Vet Immunol Immunopathol, 2001
- These results suggest that the role of amphiphiles such as SDS or arachidonic acid or of p47 phox  [ **NCF1** ]  phosphorylation in the elicitation of NADPH oxidase activation could be to disrupt the p40 phox  [ **NCF4** ]  p47 phox  [ **NCF1** ]  complex rather than to break an intramolecular interaction in p47 phox  [ **NCF1** ] .  Ref: 11258927 Biochemistry, 2001
- Thus p40 phox  [ **NCF4** ]  participates in activation of the phagocyte oxidase by regulating membrane recruitment of p67 phox and p47 phox  [ **NCF1** ]  via the PB1 PC interaction with p67 phox.  Ref: 12456638 EMBO J, 2002
- Upon stimulation, tri phox cytosolic components of NADPH oxidase p40 phox  [ **NCF4** ] , p47 phox  [ **NCF1** ]  and p67 phox bind to actin filaments.  Ref: 11333112 Redox Rep, 2001
- We also show that an Arg to Gln mutation in the PX domain of p47phox  [ **NCF1** ] , which is found in patients with chronic granulomatous disease, eliminates phosphoinositide binding, as does the analogous mutation in the PX domain of p40phox  [ **NCF4** ] .  Ref: 11433300 Nat Cell Biol, 2001

**Non-interactive relationship** (e.g. studied together, co-existance, homology, etc.)

- Unlike p47 phox  [ **NCF1** ] , the association of p40 phox  [ **NCF4** ]  with cytoskeleton was not induced by the PMA stimulation.  Ref: 11056390 J Biochem Tokyo , 2000
- The phox phagocyte oxidase homology PX domain occurs in the mammalian phox proteins p40 phox  [ **NCF4** ]  and p47 phox  [ **NCF1** ] , the polarity establishment protein Bem1p in budding yeast, and a variety of proteins involved in membrane trafficking.  Ref: 11563857 Biochem Biophys Res Commun, 2001
- Using reverse transcriptase polymerase chain reaction and rapid amplification of cDNA ends, we cloned and sequenced the full length cDNAs for five bison NADPH oxidase components p22 phox, p40 phox  [ **NCF4** ] , p47 phox  [ **NCF1** ]  and p67 phox, and gp91 phox.  Ref: 12223206 Comp Biochem Physiol B Biochem Mol Biol, 2002
- The NADPH oxidase complex of phagocytes comprises a membrane associated flavocytochrome b559, and 4 cytosolic components p47phox  [ **NCF1** ] , p67phox, p40phox  [ **NCF4** ] , and the small GTPase Rac.  Ref: 12875368 Inflammation, 2003
- Activation of the phagocyte NADPH oxidase complex requires the assembly of the cytosolic factors p47 PHOX  [ **NCF1** ] , p67 PHOX, p40 PHOX  [ **NCF4** ] , and Rac1 or Rac2, with the membrane bound cytochrome b 558.  Ref: 11248021 Proc Natl Acad Sci U S A, 2001
- As with the neutrophil NADPH oxidase, the B lymphocyte NADPH oxidase consists of a membrane bound flavocytochrome b and regulatory factors including Rac and the cytosolic phox protein triad p67phox, p47phox  [ **NCF1** ] , and p40phox  [ **NCF4** ] .  Ref: 11573965 Biochem Biophys Res Commun, 2001
- Membrane binding mechanisms of the PX domains of NADPH oxidase p40phox  [ **NCF4** ]  and p47phox  [ **NCF1** ] .  Ref: 12556460 J Biol Chem, 2003
- Two cytosolic components of NADPH oxidase, p40 phox  [ **NCF4** ]  and p47 phox  [ **NCF1** ] , contain PX domains.  Ref: 12556460 J Biol Chem, 2003
- Further studies on the full length p40 phox  [ **NCF4** ]  and p47 phox  [ **NCF1** ]  proteins showed that an intramolecular interaction between the C terminal Src homology 3 domain and the PX domain prevents the nonspecific monolayer penetration of p47 phox  [ **NCF1** ] .  Ref: 12556460 J Biol Chem, 2003
- Whereas phosphorylated p40PHOX  [ **NCF4** ]  alone did not bind to cytochrome b558, phosphorylated p47PHOX  [ **NCF1** ]  did, and, moreover, it allowed the binding of p40PHOX  [ **NCF4** ]  to the cytochrome.  Ref: 11917128 Proc Natl Acad Sci U S A, 2002
- Comparison of the deduced amino acids showed that dolphin p40 phox  [ **NCF4** ]  sequence shared 88.8% similarity with the human p40 phox  [ **NCF4** ] , that dolphin p47 phox  [ **NCF1** ]  sequence shared 87.7% similarity with the bovine p47 phox  [ **NCF1** ] ,.  Ref: 11182145 Vet Immunol Immunopathol, 2001
- Additionally, phosphoinositide binding to the PX domains of p40 phox  [ **NCF4** ]  and p47 phox  [ **NCF1** ]  appears to play a critical role in the active assembly of the neutrophil oxidase complex.  Ref: 11884510 J Cell Sci, 2002
- The superoxide generating NADPH oxidase complex of phagocytes consists of a membranal heterodimeric flavocytochrome cytochrome b 559, composed of gp91 phox and p22 phox subunits, and four cytosolic proteins, p47 phox  [ **NCF1** ] , p67 phox, p40 phox  [ **NCF4** ] , and the small GTPase Rac 1 or 2.  Ref: 11733522 J Biol Chem, 2002
- The other components of the respiratory burst oxidase are water soluble proteins of cytosolic origin, namely p67phox, p47phox  [ **NCF1** ] , p40phox  [ **NCF4** ]  and Rac.  Ref: 12440767 Cell Mol Life Sci, 2002
- The NADPH oxidase of phagocytic cells is regulated by the cytosolic factors p47 phox  [ **NCF1** ] , p67 phox, and p40 phox  [ **NCF4** ]  as well as by the Rac1 Rho GDI heterodimer.  Ref: 11258927 Biochemistry, 2001
- The superoxide generating NADPH oxidase complex of phagocytes consists of a membrane associated flavocytochrome b 559 and four cytosolic components as follows p47 phox  [ **NCF1** ] , p67 phox, p40 phox  [ **NCF4** ] , and the small GTPase Rac 1 or 2.  Ref: 12475976 J Biol Chem, 2003
- Here we show that the PX domains of p40 phox  [ **NCF4** ]  and p47 phox  [ **NCF1** ]  directly bind to phosphoinositides p40 phox  [ **NCF4** ]  prefers Ptdlns 3 P, while p47 phox  [ **NCF1** ]  does Ptdlns 4 P and Ptdlns P 2.  Ref: 11563857 Biochem Biophys Res Commun, 2001
- The p40phox  [ **NCF4** ]  and p47phox  [ **NCF1** ]  PX domains of NADPH oxidase target cell membranes via direct and indirect recruitment by phosphoinositides.  Ref: 11729195 J Biol Chem, 2002
- From the comparison of the presence or absence of interaction with various truncated forms of the proteins, we confirmed that the SH3 domain of p40 phox  [ **NCF4** ]  interacts with the C terminal proline rich region of p47 phox  [ **NCF1** ] .  Ref: 11258927 Biochemistry, 2001
- It consists of membranous cytochrom b558 composed of gp91 phox and p22 phox, and four cytosolic components, p47 phox  [ **NCF1** ] , p67 phox, rac p21 and p40 phox  [ **NCF4** ] , which translocate to the membrane upon activation.  Ref: 10695016 Hum Cell, 1999
- The activators p67 phox and p47 phox  [ **NCF1** ]  form a ternary complex together with p40 phox  [ **NCF4** ] , an adaptor protein with unknown function, comprising the PX PB2, SH3 and PC motif containing domains p40 phox  [ **NCF4** ]  associates with p67 phox via binding of the p40 phox  [ **NCF4** ]  PC motif to the p67 phox PB1 domain, while p47 phox  [ **NCF1** ]  directly interacts with p67 phox but not with p40 phox  [ **NCF4** ] .  Ref: 12456638 EMBO J, 2002
- These findings, in combination with earlier reports, allow us to propose a model for the architecture of the resting complex in which p67 phox acts as the bridging molecule that connects p40 phox  [ **NCF4** ]  and p47 phox  [ **NCF1** ] .  Ref: 11796733 J Biol Chem, 2002
- RT PCR with specific primers revealed that dolphin neutrophils expressed the mRNAs of the major NADPH oxidase components, which included membrane associated flavocytochrome b gp91 phox and p22 phox and cytosolic factors p40 phox  [ **NCF4** ] , p47 phox  [ **NCF1** ] , and p67 phox, implying the existence of these protein homologues in dolphin neutrophils.  Ref: 10980319 Dev Comp Immunol, 2001
- OBJECTIVE Superoxide generating NADPH oxidase consists of the membrane bound cytochrome b558 gp91phox and p22Phox and the cytosolic components p67phox, p47phox  [ **NCF1** ] , p40phox  [ **NCF4** ]  and rac.  Ref: 11339504 Inflamm Res, 2001
- In phagocytes and in Epstein Barr virus immortalized B lymphocytes, NADPH oxidase generates O 2 through an electron transfer chain the activity of which depends on the assembly of three, p67 phox, p47 phox  [ **NCF1** ]  and p40 phox  [ **NCF4** ]  cytosolic activating factors with Rac 1 2 and a membrane redox component, cytochrome b 558.  Ref: 10973811 Biochem Biophys Res Commun, 2000
- Activation of the phagocyte NADPH oxidase complex requires assembly of the cytosolic factors p47PHOX  [ **NCF1** ] , p67PHOX, p40PHOX  [ **NCF4** ] , and Rac with the membrane bound cytochrome b558.  Ref: 11917128 Proc Natl Acad Sci U S A, 2002
- Reconstitution of oxidase activity was performed in vitro by using a heterologous cell free assay consisting of membrane suspended or solubilized and purified cytochrome b558 that was associated with cytosol or with the isolated cytosolic activating factors p67 phox, p47 phox  [ **NCF1** ] , p40 phox  [ **NCF4** ]  from healthy or CGD patients.  Ref: 10672014 Eur J Biochem, 2000
- it was shown to be involved in both assembly and activation of oxidase complex while p47 phox  [ **NCF1** ]  proceeded as a positive effector and increased the affinity of p67 phox with cytochrome b 558, and p40 phox  [ **NCF4** ]  stabilizes the resting state.  Ref: 10924123 Biochemistry, 2000
- NADPH oxidase activity depends on the assembly of the cytosolic activating factors, p67 phox, p47 phox  [ **NCF1** ] , p40 phox  [ **NCF4** ] , and Rac with cytochrome b 558.  Ref: 10924123 Biochemistry, 2000
- The sequences of these cDNAs showed that dolphin p40 phox  [ **NCF4** ] , p47 phox  [ **NCF1** ]  and p67 phox clones contained open reading frames encoding predicted polypeptides of 33 91 and 526 amino acids, respectively.  Ref: 11182145 Vet Immunol Immunopathol, 2001
- Furthermore, PKD localizes to the area of particle intake in the cell and phosphorylates two of the three cytosolic components of the NADPH oxidase, p40 phox  [ **NCF4** ]  and p47 phox  [ **NCF1** ] .  Ref: 11903052 Biochem J, 2002
- Small angle neutron scattering and gel filtration analyses of neutrophil NADPH oxidase cytosolic factors highlight the role of the C terminal end of p47phox  [ **NCF1** ]  in the association with p40phox  [ **NCF4** ] .  Ref: 11258927 Biochemistry, 2001
- We investigated whether VSMCs from human resistance arteries HVSMCs possess a functionally active, angiotensin II Ang II regulated NAD P H oxidase that contains neutrophil oxidase subunits, including p22phox, gp91phox, p40phox  [ **NCF4** ] , p47phox  [ **NCF1** ] , and p67phox.  Ref: 12065324 Circ Res, 2002
- A. phagocytophila decreased levels of p22 phox, but not gp91 phox, p47 phox  [ **NCF1** ] , p67 phox, or P40 phox  [ **NCF4** ]  reactive with each component specific antibody in human peripheral blood neutrophils and HL 60 cells.  Ref: 11854221 Infect Immun, 2002
- The bottle nosed dolphin NADPH oxidase cytosolic components, p40 phox  [ **NCF4** ] , p47 phox  [ **NCF1** ]  and p67 phox cDNA s were cloned from mitogen stimulated peripheral white blood cell mRNA utilizing the reverse transcription polymerase chain reaction.  Ref: 11182145 Vet Immunol Immunopathol, 2001
- Molecular cloning and identification of bottle nosed dolphin p40 phox  [ **NCF4** ] , p47 phox  [ **NCF1** ]  and p67 phox.  Ref: 11182145 Vet Immunol Immunopathol, 2001
- mRNA expression of superoxide dismutases, glutathione peroxidase, and NAD P H oxidase subunits gp91phox, p40phox  [ **NCF4** ] , p47phox  [ **NCF1** ] , and p67phox remained unchanged.  Ref: 11834532 Arterioscler Thromb Vasc Biol, 2002
- The superoxide O 2 generating NADPH oxidase complex of phagocytes consists of a membrane associated flavocytochrome cytochrome b 559 and four cytosolic proteins, p47 phox  [ **NCF1** ] , p67 phox, p40 phox  [ **NCF4** ] , and the small GTPase Rac Rac1 or 2.  Ref: 11007780 J Biol Chem, 2000
- This deficiency may have resulted from a gene mutation and or abnormality of the NADH oxidase components, particularly cytochrome b558 gp91phox and p22phox in membrane and cytosol factors p47phox  [ **NCF1** ] , p67phox, p40phox  [ **NCF4** ]  and others in neutrophil.  Ref: 12145629 Nippon Ishinkin Gakkai Zasshi, 2002
- Our small angle neutron scattering experiments show that p40 phox  [ **NCF4** ]  binds to the full length p47 phox  [ **NCF1** ]  in solution in the absence of phosphorylation.  Ref: 11258927 Biochemistry, 2001
- To identify the MP binding oxidase subunit three different binding assays were carried out utilizing free or immobilized recombinant p47 phox  [ **NCF1** ] , p67 phox, p40 phox  [ **NCF4** ]  and Rac1 in conjunction with immobilized MP or soluble 125 I tyr MP, respectively.  Ref: 11301039 Biochem Pharmacol, 2001
- Western blot analysis using anti human p40 phox  [ **NCF4** ] , p47 phox  [ **NCF1** ]  and p67 phox antibodies demonstrated that dolphin neutrophil possesses p40 phox  [ **NCF4** ] , p47 phox  [ **NCF1** ]  and p67 phox with similar molecular masses and structures, to each counterpart in human neutrophils, except for the p67 phox COOH terminus.  Ref: 11182145 Vet Immunol Immunopathol, 2001
- Therefore, the p40 phox  [ **NCF4** ]  and p47 phox  [ **NCF1** ]  PX domains can target subcellular membranes via direct or indirect recruitment by phosphoinositides, while both are under the control of phosphatidylinositol 3 kinase activity.  Ref: 11729195 J Biol Chem, 2002
- In addition, SH3 domains of p47 phox  [ **NCF1** ]  and p40 phox  [ **NCF4** ]  and the carboxyterminal SH3 domain of p67 phox exhibited a low affinity towards MP.  Ref: 11301039 Biochem Pharmacol, 2001
- Co immunoprecipitation studies and measurement of NADPH dependent reactive oxygen species production cytochrome c reduction assay demonstrated that p22 phox, gp91 phox, p47 phox  [ **NCF1** ] , p67 phox, and p40 phox  [ **NCF4** ]  existed as a functional complex in the cytoskeletal fraction.  Ref: 11893732 J Biol Chem, 2002
- After subcellular protein fractionation, approximately 50% of the gp91 phox 91 kDa band, p22 phox, p67 phox, and p40 phox  [ **NCF4** ]  pools and approximately 30% of the p47 phox  [ **NCF1** ]  were present in the 1475 x g nucleus rich fraction.  Ref: 11893732 J Biol Chem, 2002
- p47 phox  [ **NCF1** ] , two truncated forms of p47 phox  [ **NCF1** ] , namely, p47 phox  [ **NCF1** ]  without its C terminal end residues 1 358 and p47 phox  [ **NCF1** ]  without its N terminal end residues 147 390, and p40 phox  [ **NCF4** ]  were found to be monomeric in solution.  Ref: 11258927 Biochemistry, 2001
- A number of cytosolic proteins are involved in its activation deactivation p47phox  [ **NCF1** ] , p67phox, p40phox  [ **NCF4** ]  and the small GTP binding protein, rac.  Ref: 11716484 Biochem Biophys Res Commun, 2001
